# Combined transcriptome and widely targeted metabolome analysis reveals the potential mechanism of HupA biosynthesis and antioxidant activity in *Huperzia serrata*

**DOI:** 10.3389/fpls.2024.1411471

**Published:** 2024-06-17

**Authors:** Hao Wu, Yu Shen, Fen Zou, Shiqing Yao, Yaling Chen, Huilin Yang, Xiangdong Luo

**Affiliations:** College of Life Science, Jiangxi Normal University, Nanchang, China

**Keywords:** *Huperzia serrata*, biosynthesis mechanism, antioxidant activity, transcriptome, metabolome

## Abstract

**Introduction:**

*Huperzia serrata* is a traditional Chinese herb that has gained much attention for its production of Huperzine A (HupA). HupA has shown promise on treating Alzheimer's disease (AD). However, the biosynthetic pathway and molecular mechanism of HupA in *H. serrata* are still not well understood.

**Methods:**

Integrated transcriptome and metabolome analysis was performed to reveal the molecular mechanisms related to HupA biosynthesis and antioxidant activity in *Huperzia serrata*.

**Results:**

HT (*in vitro H. serrata* thallus) exhibits higher antioxidant activity and lower cytotoxicity than WH (wild *H. serrata*). Through hierarchical clustering analysis and qRT-PCR verification, 7 important enzyme genes and 13 transcription factors (TFs) related to HupA biosynthesis were detected. Among them, the average |log_2_FC| value of *CYP* (Cytochrome P450) and *CAO* (Copper amine oxidase) was the largest. Metabolomic analysis identified 12 metabolites involved in the HupA biosynthesis and 29 metabolites related to antioxidant activity. KEGG co-enrichment analysis revealed that tropane, piperidine and pyridine alkaloid biosynthesis were involved in the HupA biosynthesis pathway. Furthermore, the phenylpropanoid, phenylalanine, and flavonoid biosynthesis pathway were found to regulate the antioxidant activity of *H. serrata*. The study also identified seven important genes related to the regulation of antioxidant activity, including *PrAO* (primary-amine oxidase). Based on the above joint analysis, the biosynthetic pathway of HupA and potential mechanisms of antioxidant in *H. serrata* was constructed.

**Discussion:**

Through differential transcriptome and metabolome analysis, DEGs and DAMs involved in HupA biosynthesis and antioxidant-related were identified, and the potential metabolic pathway related to HupA biosynthesis and antioxidant in *Huperzia serrata* were constructed. This study would provide valuable insights into the HupA biosynthesis mechanism and the *H. serrata* thallus medicinal value.

## 1 Introduction

Alzheimer’s disease (AD), is a neurodegenerative disorder characterized by progressive memory and cognitive dysfunction. It has emerged as the third leading cause of death, following cancer and cardiovascular diseases (Ozben and Ozben, 2019). Recent growth data indicated that the global dementia population will triple by 2050 (Scheltens et al., 2021). AD has become a social problem that needs to be addressed urgently (Kumar et al., 2018). Currently, there are no effective medications that can prevent the pathogenesis of AD or slow its progression. However, acetylcholinesterase inhibitors (AChEI) have shown promise in alleviating the symptoms of the disease (Breijyeh and Karaman, 2020). And it has been confirmed that Huperzine A (HupA) is an efficient, highly selective and naturally reversible AChEI, with unique pharmacological activities and low toxicity (Le et al., 2020). These advantages suggest that HupA holds great potential for the treatment of AD. In fact, it has been approved by the U. S. Food and Drug Administration as a new dietary supplement for improving AD (Damar et al., 2016).

HupA is a plant-derived lycopodium alkaloid, which was first isolated from *H. serrata* by Liu et al. in 1986. Whereas, the content of HupA in wild *H. serrata* is very low, approximately 0.006% (Ferreira et al., 2014). Moreover, *H. serrata* grows slowly and reproduces difficultly under natural conditions, from spore germination to a complete plant of 12 cm high takes 6–15 years, and it cannot be cultivated on a large scale (Jiang et al., 2019; Cruz-Miranda et al., 2020). Consequently, researchers have explored alternative methods such as chemical synthesis and endophytic fermentation to obtain HupA (Han et al., 2020; Sang et al., 2020). However, the synthetic HupA exhibits low chemical activity, significant side effects, requires harsh reaction conditions, and is expensive. Moreover, the fermentation yield of endophytic bacteria is low and unstable, with the possibility of no longer producing HupA after several generations of subculture. Therefore, understanding the biosynthetic pathway and molecular mechanism of HupA in *H. serrata* is considered the most efficient and promising approach for HupA production through gene engineering biosynthesis.

The proposal of HupA biosynthetic pathway can be traced back to 2004 (Ma and Gang, 2004). The ^14^C and ^13^C labeling experiments proposed the biosynthetic pathway of lycopodium alkaloids. Since then, numerous scientists have endeavored to uncover the key enzyme genes related to HupA biosynthesis in *H. serrata*. For example, based on comparative transcriptome analysis of *H. serrata* and *Phlegmariurus carinatus*, four CYP450 transcripts most likely to be involved in HupA biosynthesis were selected (Luo et al., 2010a). Additionally, transcriptome analysis of *H. serrata* re-identified *LDC*, *CAO*, and *PKS* as important genes involved in the initiation of HupA synthesis (Luo et al., 2010b; Yang et al., 2017). Recently, Sattely research group identified neofunctionalized α-carbonic anhydrases (CAHs) as a class of key enzyme in alkaloid biosynthesis and described a series of scaffold tailoring steps that generate HupA in *Phlegmariurus tetrastichus* (Nett et al., 2023). These studies provide valuable information for revealing the molecular mechanisms on the biosynthesis and metabolism of HupA. However, the biosynthetic pathway of HupA is very complex, and the genetic background of *H. serrata* remains unclear, with limited molecular date available on its genome. Moreover, previous studies on the molecular mechanism of HupA biosynthesis only focused on wild *H. serrata* or its related wild species. And there is not significant difference in HupA content among different tissues of *H. serrata.* Therefore, it is still a challenge to establish the relationship between HupA biosynthesis and key enzyme genes.

Fortunately, our group has successfully obtained *in vitro H. serrata* thallus (HT) through the regeneration of wild *H. serrata* (WH). HT can stably subculture and accumulate HupA (Yang et al., 2021). And we found that the HupA content of HT was significantly lower than that of WH, which provided a good research material for illuminating the molecular mechanism of HupA biosynthesis. Previous studies have also shown that the biosynthetic metabolic pathway of HupA involves a large number of redox reactions and related enzyme genes (Li et al., 2022). Therefore, in this study, we conducted transcriptome, metabolomics, antioxidant analysis using WH and HT. These results will facilitate us revealing the key enzyme genes and metabolites involved in the HupA biosynthesis pathway, as well as the antioxidant activity and its interacting regulatory network in *H. serrata*. These results not only would help us to understand the molecular mechanism of HupA biosynthesis, but also provide some useful information for further investigation and utilization of HT.

## 2 Materials and methods

### 2.1 Plant materials and growth conditions

The plant materials of wild *H. serrata* (WH) used in the study were collected from Fujian province in China. Then, the fresh spore-bearing stems of WH were used for the subsequent micropropagation *in vitro*, from which original thallus of *H. serrata* (HT) was regenerated. HT can produce HupA and able to subculture stably. The culture condition of HT was maintained at 25 ± 1°C. The photoperiod was 2000 lx for 13 h daylight, and 11 h darkness.

### 2.2 Preparation of methanol extract of *H. serrata*

Isolation of the methanol extract of *H. serrata* was conducted following the method of Fagbemi et al. (2022), with slight modifications. A total of 10g of dried *H. serrata* samples were subjected to three extractions using 150 mL of methanol (analytical purity). The resulting solution was filtered using filter paper, and the extract was subsequently concentrated using a rotary evaporator to obtain a powdered extract. Finally, the extract was stored at -20°C for further experimental analysis.

### 2.3 Extraction and content determination of HupA

The extraction method of HupA in the sample followed previous reports with some modifications (Yu et al., 2010). Leaves and stems of wild *H. serrata* and *H. serrata* thallus were washed and dried in a 40°C oven for 3 days. They were then ground into a powder. Powdered samples (0.5g) were dissolved in 8 mL of 2% tartaric acid and placed in a 40-degree water bath for 24 h. After that, ultrasonic extraction was performed for 30 min, followed by centrifugation for 15 min at 5000 rpm. This process was repeated three times. The supernatant was collected and the pH was adjusted to between 9 and 10. The collected liquid was then dried at 40°C. Finally, methanol was evaporated to 1 mL. The content of HupA was detected using High-Performance Liquid Chromatography (HPLC).

### 2.4 Determination of the antioxidant capacity of *H. serrata* extracts

#### 2.4.1 DPPH radical scavenging activity assay

The DPPH radical scavenging capacities of the methanol extracts of wild *H. serrata* and *H. serrata* thallus were measured according to Mohamed et al. (2020), with some modifications. A series of 200 μL diluted sample extracts or methanol (control) were added to 1.5 mL of DPPH. The mixtures were shaken vigorously and then placed in the dark at room temperature for 30 min. Afterward, measure the absorbance at 517 nm.

#### 2.4.2 ABTS radical scavenging activity assay

The ABTS assay was performed using the method described by Tan et al. (2021) with minor modifications. First, an equal volume of potassium persulfate (2.45 mmol/L) and ABTS (7 mmol/L) were mixed to prepare the ABTS working solution. The ABTS working solution was incubated in the dark at room temperature for 12–16 h. Then, 1 mL of the ABTS working solution was diluted with 19 mL of ethanol, and the absorbance at 734 nm was 0.70 ± 0.05. Then, 1.9 mL of diluted ABTS working solution was mixed with 0.2 mL of the sample and incubated in the dark at 25°C for 6 min. Finally, the absorbance at 734 nm was measured.

#### 2.4.3 Hydroxyl radical scavenging activity assay

The determination of OH**^-^** radical scavenging activity was based on the previous method and slightly modified (Gulcin, 2020). The different concentrations of diluted extracts were mixed with 1 mL of 0.75 mM phenanthroline alcohol solution, 2 mL of phosphate buffered solution (0.2 mM PBS), 1 mL of 0.75 mM FeSO_4_, 1 mL of deionized water, and 1 mL of H_2_O_2_ solution (0.03%). respectively. The mixture was fully mixed and incubated at 37°C for 60 min. Finally, the absorbance of the reaction mixture was read at 512 nm.

#### 2.4.4 Ferric reducing antioxidant power assay

The FRAP assay was performed using the methodology described by Xia et al. (2023), with minor modification. Firstly, 300 mM acetate buffer, 25 mL 10 mM TPTZ (2,4,6-tri-pyridyl-s-triazine) solution and 2.5 mL of 20 mM FeCl_3_·6H_2_O solution were fully mixed to prepare the stock solution. 25 mL acetate buffer, 2.5 mL TPTZ solution, and 2.5 mL FeCl_3_·6H_2_O were mixed to prepare a fresh working solution and incubated at 37°C. Then 200 µL extract was mixed with 2.8mL working solution and reacted in the dark at room temperature for 30 min. The absorbance is read at 593 nm. The 200 µL distilled water was mixed with 2.8 mL working solution as the blank group.

### 2.5 Cell viability assay

Cell viability was measured according to the method of Luo et al. (2023). RAW 264.7 cells were cultured in DMEM at 37°C and 5%CO_2_. When the cells were in the logarithmic growth phase, DMEM medium was added to make the cell concentration at 1 × 10^5^/mL, and then incubated for 12 h. The medium was removed and treated with different concentrations of extract for 24 h. After treatment, 10μL MTT (5mg/mL in PBS) and 90μL DMEM were added to each well. After 4 h, the media solution was removed and 150μL DMSO was added to each well for 30 min. Finally, the solution absorbance of each well was detected by a microplate reader at 570 nm.

### 2.6 RNA extraction and RNA sequencing

Total RNA of the samples was extracted with an RNAprep Pure Plant kit (DP441, Tiangen, China). The RNA quality was assessed using a NanoPhotometer spectrophotometer (IMPLEN, CA, USA), Qubit 2.0 Fluorometer (Life Technologies, CA, USA), and Agilent Bioanalyzer 2100 system (Agilent Technologies, CA, USA). Then the cDNA library was constructed. The cDNA library was preliminarily quantified using Qubit2.0 and detected using Agilent 2100. The cDNA library was sequenced on the Illumina platform Novaseq6000 system after qualified detection (Chen et al., 2021). Illumina RNA-Seq was performed by Metware Biotechnology Co. Ltd. (Wuhan, China). After sequencing, the obtained image data was converted into a large amount of raw data by CASAVA base recognition. To obtain high-quality data, sequences with adapters were trimmed, and reads with low quality were removed.

### 2.7 *De novo* assembly and functional annotation

After obtaining high quality clean reads, use Trinity (version 2.0.6) to assemble clean data according to the default parameters for *de novo* assembly. Subsequently, Busco software was used to assess their integrity. The transcript sequences obtained from Trinity splicing were used as reference sequences for subsequent analyses. The longest Cluster sequence obtained after Corset hierarchical clustering (https://code.google.com/p/corset-project/) was used as Unigene for subsequent analyses. All assembled sequences were blasted in different databases including the Kyoto encyclopedia of genes and genomes (KEGG), NCBI non-redundant protein (Nr), Swiss-Prot, gene ontology (GO), cluster of orthologous groups of proteins (COG), eukaryotic ortholog groups (KOG), and the translation of EMBL (TrEMBL) using DIAMOND BLASTX software (Buchfink et al., 2015). After predicting the amino acid sequences of the unigenes, the annotation information of the unigenes was obtained by comparing with the protein family (Pfam) database using the HMMER software (version 3.1b2).

### 2.8 Differential gene analysis

The Trinity assembled and de-redundant transcripts were used as reference sequences, and RSEM software was used to blast the clean reads and reference sequences of each sample. FPKM was used to calculate the expression level of each gene. Differential expression analysis between sample groups was performed using DESeq2 (Love et al., 2014; Varet et al., 2016). Specified paired transcriptome comparison (WH vs HT) was performed to identify the major differential expression genes (DEGs) that had an absolute value of log_2_FC ≥ 1 and an FDR < 0.5. DEGs were annotated based on their expression levels in different samples following COG, GO, KEGG, KOG, Pfam, Swiss-Prot, TrEMBL, eggNOG, and Nr annotations.

### 2.9 Quantitative real-time PCR analysis

Purified RNA (1 µg for each sample) was reverse transcribed into first-strand cDNA using a cDNA Reverse Transcription Kit (PrimeScriptTM RT Master Mix, Takara) according to the manufacturer’s instructions. qRT-PCR was conducted using a ChamQ SYBR qPCR Master Mix kit (Vazyme) and a C1000 Touch™Thermal Cycler system (Bio-Rad). 18S rRNA was used as the internal reference gene. The relative expression levels of 12 genes in the HupA biosynthetic pathway and 20 randomly selected genes were calculated by 2-ΔΔCt method.

### 2.10 Construction of the phylogenetic tree

To perform phylogenetic tree analysis, homologous genes in the HupA biosynthetic pathway were identified within the gene libraries of *Huperzia asiatica* and *Diphasiastrum complanatum* (Li et al., 2024). Subsequently, the phylogenetic tree was constructed using Mega 7.0 software, employing the highest-confidence adjacency method, specifically the neighbor-joining (NJ) algorithm.

### 2.11 Analysis of widely targeted metabolic profiling

To study the metabolite variations among wild and cultivated variety of *H. serrata*. Each group of samples was subjected to three biologically repeated metabolic analyses. For comprehensive metabolite analysis, samples were sent to Metware Biotechnology Ltd. (Wuhan, China). Mass spectrometry data of metabolites were processed using version 1.6.3 software. The variation between metabolites of two different samples was visualized by maximizing the difference between the metabolites by OPLS-DA (Orthogonal Projection-Discriminant Analysis of Latent Structures) to identify differential metabolites. After that, metabolites with VIP > 1.0 or Fold change ≥ 2 and Fold change ≤ 0.5 were selected as differential metabolites for further screening. Annotation and display of differential metabolites using the KEGG database (Kanehisa and Goto, 2000). Other analyses included Principal Component Analysis (PCA), K-means, and pathway enrichment, which were performed using R software.

### 2.12 Combined transcriptome and metabolome analyses

A comprehensive analysis was conducted to examine both differentially expressed genes and differentially accumulated metabolites. Pearson’s correlation analysis was performed for genes and metabolites detected in WH vs HT group. Results with correlation coefficients > 0.8 and p-values < 0.05 were selected for KEGG enrichment analysis. By analyzing the co-enrichment pathway maps of WH vs HT, we identified the major enrichment pathways and identified the differential genes and metabolites within these pathways.

### 2.13 Statistical analysis

All experimental data were expressed as mean ± standard deviation with three biological replicates. Differences between transcriptomic data and metabolic profiling were analyzed using SPSS 13.0 software (SPSS, USA). *P* < 0.05 indicated a significant difference. Pearson correlations between structural genes and SaMYBs were analyzed based on the cor function of the R software (www.r-project.org/) and the correlation network was visualized using Cytoscape v3.8.2.

## 3 Results

### 3.1 Description of two types of *H. serrata* and HupA content

**Figure 1:**
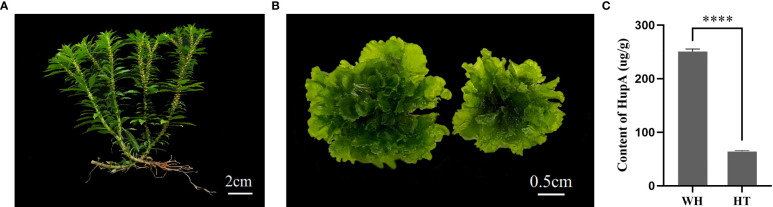
The two types of *H. serrata* used in this study. **(A)** wild *H. serrata*. **(B)** *H. serrata* thallus. **(C)** The content of HupA in WH and HT. WH and HT represents wild *H. serrata* and *H. serrata* thallus, respectively. ****p < 0.0001.

Wild *H. serrata* (WH) is a slow-growing, dark green perennial fern plant. From spore germination, a WH plant with a height of 12cm need for 6–10 years. Its vegetative organs consist of roots, stems, and leaves. The stem covered by cutinized cuticle and the cortex contains chloroplasts, which enable photosynthesis. Small spores are often found at the top. The leaves have spiral and elliptic-lanceolate shape (**Figure 1A**). However, the morphological characteristics of *in vitro H. serrata* thallus (HT, **Figure 1B**) shows significant differences from that of WH. Currently, HT does not have normal morphology of roots, stems, and leaves. Therefore, HT can only proliferate and preserve *in vitro* conditions now. But it grows much faster than WH. After 70 d of HT subculture, the diameter of the thallus was to 2.35 ± 0.2 cm, which is much larger than the leaves of WH (**Figure 1B**). Analysis of chemical component showed that the content of HupA in HT was 64.12 ± 3.24 μg·g^-1^, which was about one fourth of that in WH (250.34 ± 1.51 μg·g^-1^) (**Figure 1C**).

### 3.2 The difference of antioxidant activity between two types of *H. serrata*

**Figure 2:**
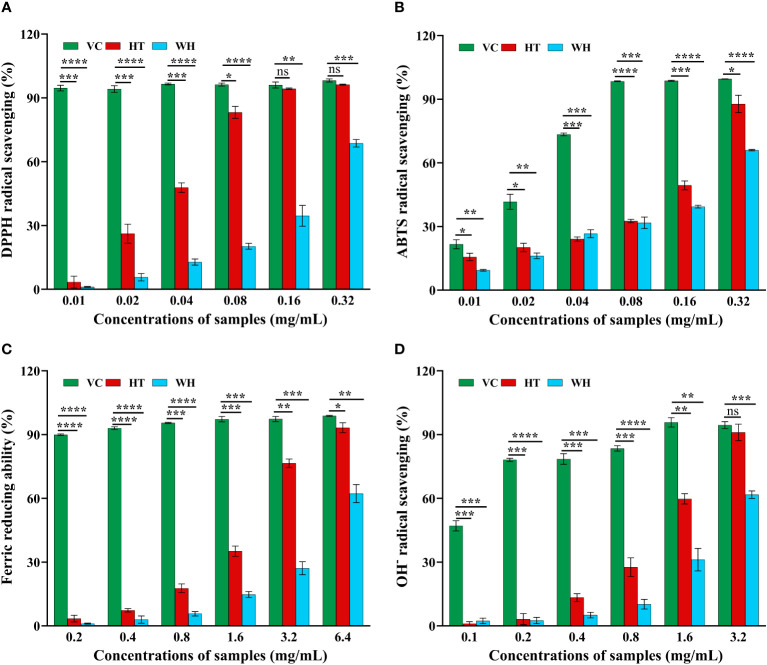
Antioxidant activity of the two *H. serrata* extracts. **(A)** DPPH radical scavenging assay. **(B)** ABTS radical scavenging assay. **(C)** Fe^3+^ reductive power assay. **(D)** Hydroxyl radical scavenging assay. VC group: Vitamin C was added as a positive control group. HT represents the extracts of *H. serrata* thallus. WH represents the extracts of wild *H. serrata*. *p<0.05, **p < 0.01, ***p < 0.001 and ****p < 0.0001.

The scavenging capacity of DPPH, ABTS, OH^−^ and FRAP were important indexes of the antioxidant capacity of the samples (Gulcin, 2020). The DPPH and ABTS scavenging ability of *H. serrata* was showed in **Figures 2A, B**, respectively. The scavenging ability showed a positive correlation with the experimental concentration. At a concentration of 0.32 mg/mL, the DPPH and ABTS scavenging activities of HT were 96.29 ± 0.25% and 87.81 ± 4.07%, respectively, which were higher than that of WH (68.69 ± 1.74% and 65.99 ± 0.33%) (**Figures 2A**, **B**). In addition, the IC_50_ values of DPPH and ABTS scavenging capacity of HT were 0.0274 mg/mL and 0.1173 mg/mL, respectively, while those of WH were 0.1121 mg/mL and 0.1896 mg/mL, respectively (**Table 1**). These data indicated that the DPPH and ABTS scavenging ability of HT was greater than that of WH.

**Table 1:** IC_50_ value of the two *H. serrata*.

Assay method	IC50 value	
	HT (mg/ml)	WH (mg/ml)
DPPH	0.02739	0.11210
ABTS^+^	0.11730	0.18960
FRAP	1.97800	5.40300
OH^-^	1.28700	2.68600

The hydroxyl radical (OH^−^) scavenging activity and the ability to reduce Fe^3+^ of WH and HT were shown in **Figures 2C, D**. The results indicated that HT had significantly higher OH^−^ scavenging activity and ability to reduce Fe^3+^ compared to WH at the same concentration. At the concentration of 3.2 mg/mL, the ability to reduce Fe^3+^ and OH^-^scavenging capacity of HT were 76.55 ± 1.95% and 91.08 ± 3.88%, respectively. And WH was 27.13 ± 3.07%, and 61.76 ± 1.76% (**Figures 2C**, **D**). Moreover, the IC_50_ values of HT were lower than those of WH (**Table 1**).

### 3.3 The difference in the effects of two *H. serrata* extracts on cell viability

**Figure 3:**
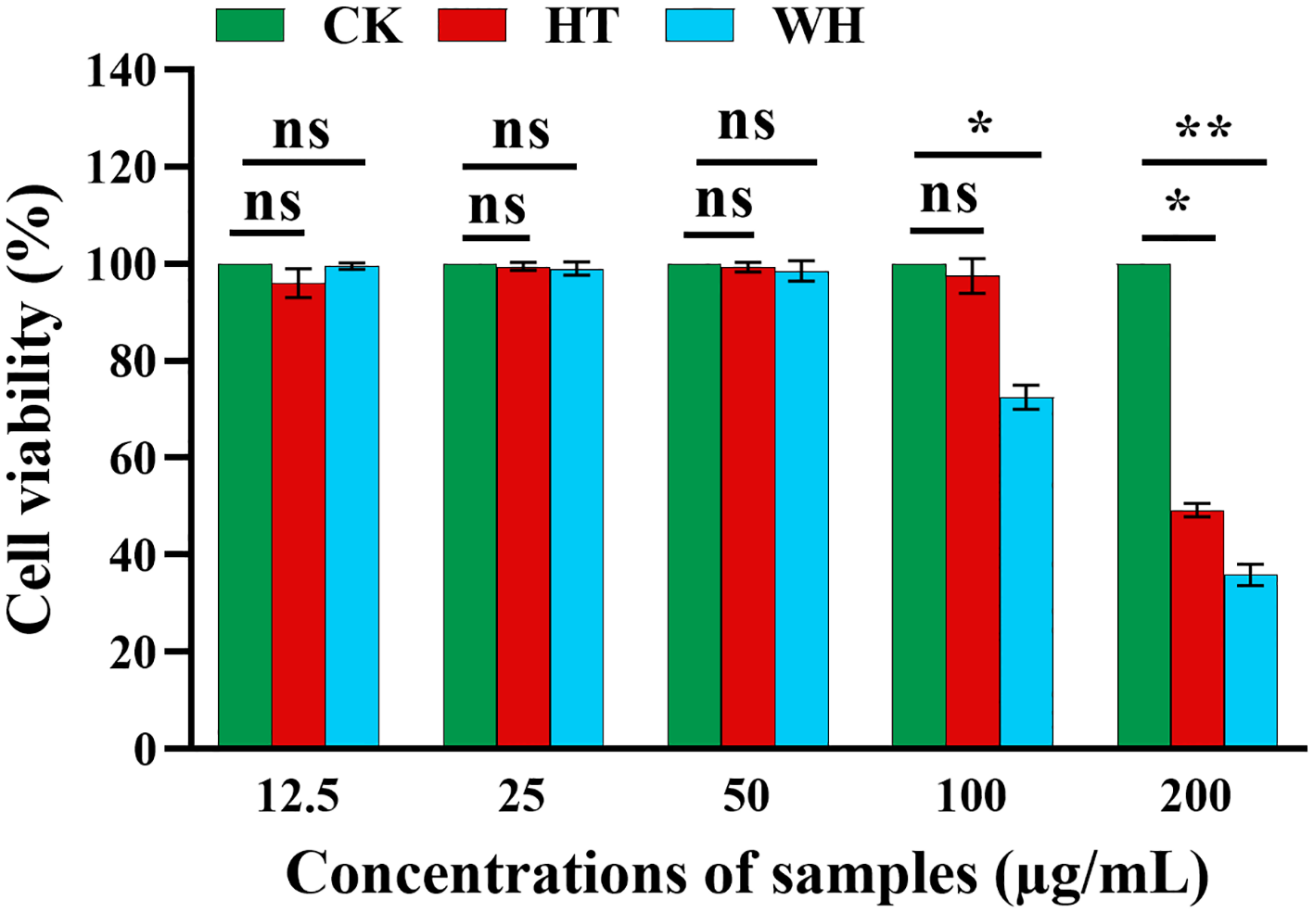
Effects of methanol extracts of two *H. serrata* on the viability of RAW264.7 cells, *p<0.05, **p < 0.01 and ***p < 0.001. CK represents the blank control group. HT represents the extracts of *H. serrata* thallus. WH represents the extracts of wild *H. serrata*.

In order to evaluate the potential cytotoxicity of WH and HT, the effects of methanol extracts of WH and HT on the viability of RAW264.7 cells were detected by MTT assay. As shown in **Figure 3**, when the concentration was ≤ 50 μg/mL, the methanol extracts of WH and HT had no significant effect on cell viability (both > 95%). However, when the concentration was 100 μg/mL, the cell survival rate of the WH group (72.49 ± 0.02%) was significantly lower than that of the HT group (97.67 ± 0.04%), indicating that the cytotoxicity of the methanol extract from WH was higher than that of HT.

### 3.4 Analysis of transcriptome differences between two types of *H. serrata*

Illumina HiSeq paired-end sequencing technology was used to analyze the transcriptome of *H. serrata* samples (WH and HT). In this study, a total of 6 cDNA libraries (WH-1, WH-2, WH-3, HT-1, HT-2, HT-3) were prepared and analyzed. The clean data of each sample reached 18 Gb, and low-quality reads were filtered out to obtain clean reads ranged from 42,468,530 to 70,379,112. Data quality Q_30_ is greater than 93% (**Supplementary Table S1**). A total of 80,523 unigenes were generated after sequence assembly. Most of the sequence lengths are distributed in 1000–2000 and ≥ 2000 (**Supplementary Figure S1**). In addition, these unigenes were searched in seven public databases (KEGG, NR, Swiss-Prot, TrEMBL, KOG, GO and Pfam), and 87% of the unigenes were annotated by at least one database (**Supplementary Figure S2**). In summary, RNA-seq data is reliable and of high quality, which can be used for subsequent research.

Then, the differential genes (DEGs) were identified by comparing the FPKM values of each gene (|log_2_FC| ≥ 1 and FDR < 0.05) in WH and HT. A total of 80,379 DEGs were detected in WH vs HT, of which 36,982 were down-regulated and 43,397 were up-regulated. These DEGs were annotated to 134 KEGG pathways, among which enriched pathways included tropane, piperidine and pyridine alkaloid biosynthesis (ko00960), phenylalanine metabolism (ko00360), phenylpropanoid biosynthesis (ko00940), etc. The results indicated there were a large number of DEGs between WH and HT, providing valuable genetic resources for studying specific traits.

### 3.5 Analysis of metabolome differences between two types of *H. serrata*

Metabolites in WH and HT were isolated and identified based on ultra-high performance liquid chromatography-tandem mass spectrometry (UPLC-MS/MS) (Wang et al., 2011). In this study, a total of 1374 metabolites were detected. The PCA analysis results showed that PC1 and PC2 accounted for 84.72% and 4.2% of the total variance, respectively (**Supplementary Figure S3**). These results demonstrate the good repeatability of each sample group and significant differences between the groups, making them suitable for subsequent data analysis.

**Figure 4:**
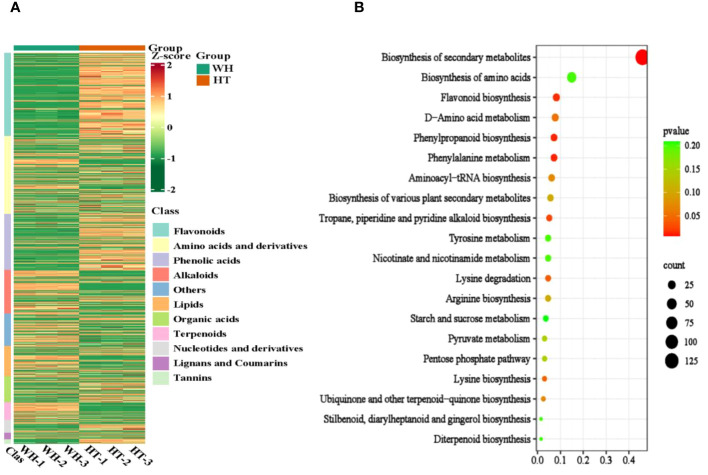
Analysis of total DAMs. **(A)** Heat map of total DAMs. **(B)** KEGG enrichment bubble diagram of total DAMs. WH and HT represents wild *H. serrata* and *H. serrata* thallus, respectively.

To gain insights into the variance of metabolites among WH and HT, the differentially accumulated metabolites (DAMs) were selected based on the criteria of FC ≥ 2 or ≤ 0.5 and *P* < 0.05. A total of 925 DAMs were detected, of which 391 were down-regulated and 534 were up-regulated. These DAMs were classified into 11 categories, including amino acids and derivatives (184), alkaloids (103), flavonoids (197), lignans and coumarins (15), lipids (72), nucleotides and derivatives (31), organic acids (61), phenolic acids (133), terpenoids (42), tannins (10) and others (77) (**Supplementary Table S2**). And the contents of these DAMs in WH and HT were significantly different (**Figure 4A**). In addition, these DAMs were annotated to a total of 87 pathways in the KEGG database, of which the most significantly enriched pathways were phenylalanine metabolism (ko00360), phenylpropanoid biosynthesis (ko00940), flavonoid biosynthesis (ko00941), tropane, piperidine and pyridine alkaloid biosynthesis (ko00960) and lysine degradation (ko00310) (**Figure 4B**).

### 3.6 Differentially expressed genes and TFs in HupA biosynthesis pathway

**Figure 5:**
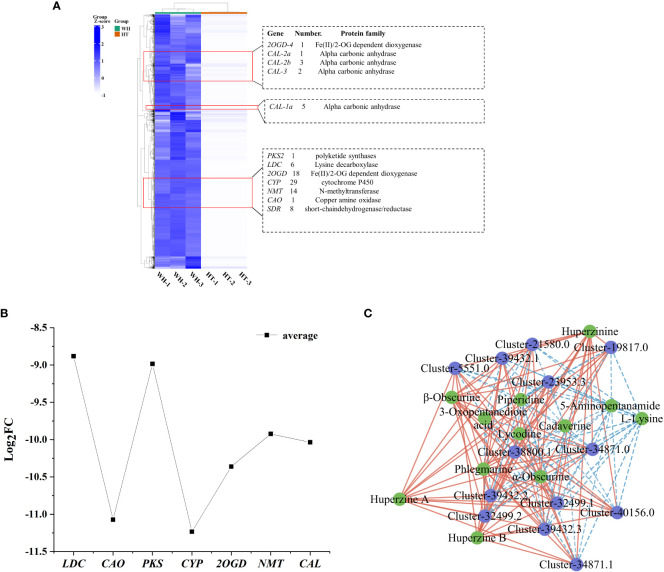
Analysis of DEGs and TFs related to HupA biosynthesis in HT and WH. **(A)** Cluster heatmap of all down-regulated DEGs in HT. **(B)** Mean values of log_2_FC for 7 enzymes. **(C)** Correlation network diagram of TFs and DAMs. The blue circles represent genes and the green circles represent metabolites. Red and blue lines indicate significant positive and negative correlations. WH and HT represents wild *H. serrata* and *H. serrata* thallus, respectively.

Cluster analysis is a valuable method for identifying genes associated with HupA biosynthesis. Therefore, combined with the reported HupA synthesis pathway in *H. serrata* and in *P. tetrastichus* (Li et al., 2022; Nett et al., 2023), we performed a cluster heatmap analysis of a total of 36,982 down-regulated DEGs. The results showed that there were three significantly enriched gene clusters. These clusters encompassed all the genes involved in HupA synthesis pathway of *H. serrata* as well as the key enzyme genes in *P. tetrastichus* (**Figure 5A**), including seven enzyme gene families, namely polyketide synthase (*PKS*), lysine decarboxylase (*LDC*), Fe(II)/2-OG dependent dioxygenase (*2OGD*), cytochrome P450 (*CYP*), N-methyltransferase (*NMT*), copper amine oxidase (*CAO*) and alpha carbonic anhydrase (*CAL*). Among them, the largest DEGs were *CAO* and *CYP*. Their average |log_2_FC| were 11.07 and 11.23, respectively, indicating that *CYP* and *CAO* were the most significant DEGs (**Figure 5B**). Then we performed qRT-PCR verification on these seven enzyme genes and other randomly selected DEGs (**Supplementary Figures S4**, **S5**). The results showed that the expression levels of these seven enzyme genes in WH were higher than HT, which was consistent with the transcriptome results (FPKM) and positively correlated with HupA content. This indicates that they are important genes involved in HupA biosynthesis, and also indicates that the transcriptome data in this study are accurate and reliable.

In order to visualize the genetic relationship of *H. serrata*, a phylogenetic tree and motif analysis were constructed. Fifty-five homologous genes (**Supplementary Table S3**) were analyzed in the gene libraries of *Huperzia asiatica* and *Diphasiastrum complanatum* (Li et al., 2024). The phylogenetic tree was constructed for each enzyme and its homologous genes, which was shown in the (**Supplementary Figures S6–S10**). The results indicated that *H. serrata* showed closer homology with *Huperzia asiatica* and more distant homology with *Diphasiastrum complanatum*. And these homologous genes have similar motif structures (**Supplementary Figures S6–S10**).

In order to study the transcription factors (TFs) that regulate the HupA biosynthesis pathway, all the TFs in WH and HT were analyzed. In this study, a total of 1826 TFs were detected in *H. serrata*. The most abundant TFs were bHLH, C3H, C2H2 and bZIP, which were 76, 98, 82 and 80, respectively (**Supplementary Figure S11**). Previous studies have shown that the biosynthesis of HupA begins with the metabolism of lysine (Yang et al., 2017). In this study, 43 TFs were involved in lysine degradation pathway according to KEGG pathway enrichment analysis. Among them, 13 TFs were annotated as histone lysine N-methyltransferases in the NR database. Then, the correlation between these 13 TFs and the 13 DAMs involved in HupA biosynthesis was analyzed. The results showed that these TFs were significantly correlated with metabolites in the HupA biosynthetic pathway, especially closely related to huperzine B, β-obscurine, hupA, huperzinine, phlegmarine, α-obscurine and lycodine (P-value < 0.01, correlation > 0.9). This indicates that these are potential TFs that regulate the HupA biosynthesis pathway (**Figure 5C**).

### 3.7 Combined transcriptome and metabolome analysis of HupA biosynthesis pathway in *H. serrata*

A total of 12 metabolites involved in the HupA biosynthesis pathway were detected in the metabolomics data, of which 7 were down-regulated metabolites in HT, while other 5 were up-regulated (**Table 2**). It is almost the same as the 15 metabolites in the HupA biosynthesis pathway reported by Li et al (Li et al., 2022). 4-(2-piperidyl)-acetoacetic acid (4PAA), pelletierine and lycodane in HupA biosynthesis pathway were not detected in WH and HT, which was possibly due to their low content or short half-life. The results of log_2_FC values showed that the main DAMs involved in the HupA biosynthesis in *H. serrata* were huperzine B, β-obscurine, hupA, huperzinine, phlegmarine, α-obscurine, and lycodine. In addition, among these metabolites, l-lysine, cadaverine and piperidine were annotated to the pathway of tropane, piperidine and pyridine alkaloid biosynthesis (ko00960).

**Table 2:** Detected metabolites involved in the biosynthetic pathway of HupA.

Compounds	Formula	Class I	WH vs HT Log_2_FC
Huperzine B	C_16_H_20_N_2_O	Alkaloids	-19.45
3-Oxopentanedioic acid	C_5_H_6_O_5_	Organic acids	0.96
β-Obscurine	C_17_H_24_N_2_O	Alkaloids	-17.98
HupA	C_15_H_18_N_2_O	Alkaloids	-19.20
Huperzinine	C_17_H_22_N_2_O	Alkaloids	-5.17
5-Aminopentanamide	C_5_H_12_N_2_O	Alkaloids	13.05
Phlegmarine	C_16_H_30_N_2_	Alkaloids	-20.19
α-Obscurine	C_17_H_26_N_2_O	Alkaloids	-18.99
L-Lysine	C_6_H_14_N_2_O_2_	Amino acids and derivatives	3.94
Cadaverine	C_5_H_14_N_2_	Alkaloids	2.02
Piperidine	C_5_H_11_N	Alkaloids	1.17
Flabellidine	C_18_H_28_N_2_O	Alkaloids	-8.34

**Figure 6:**
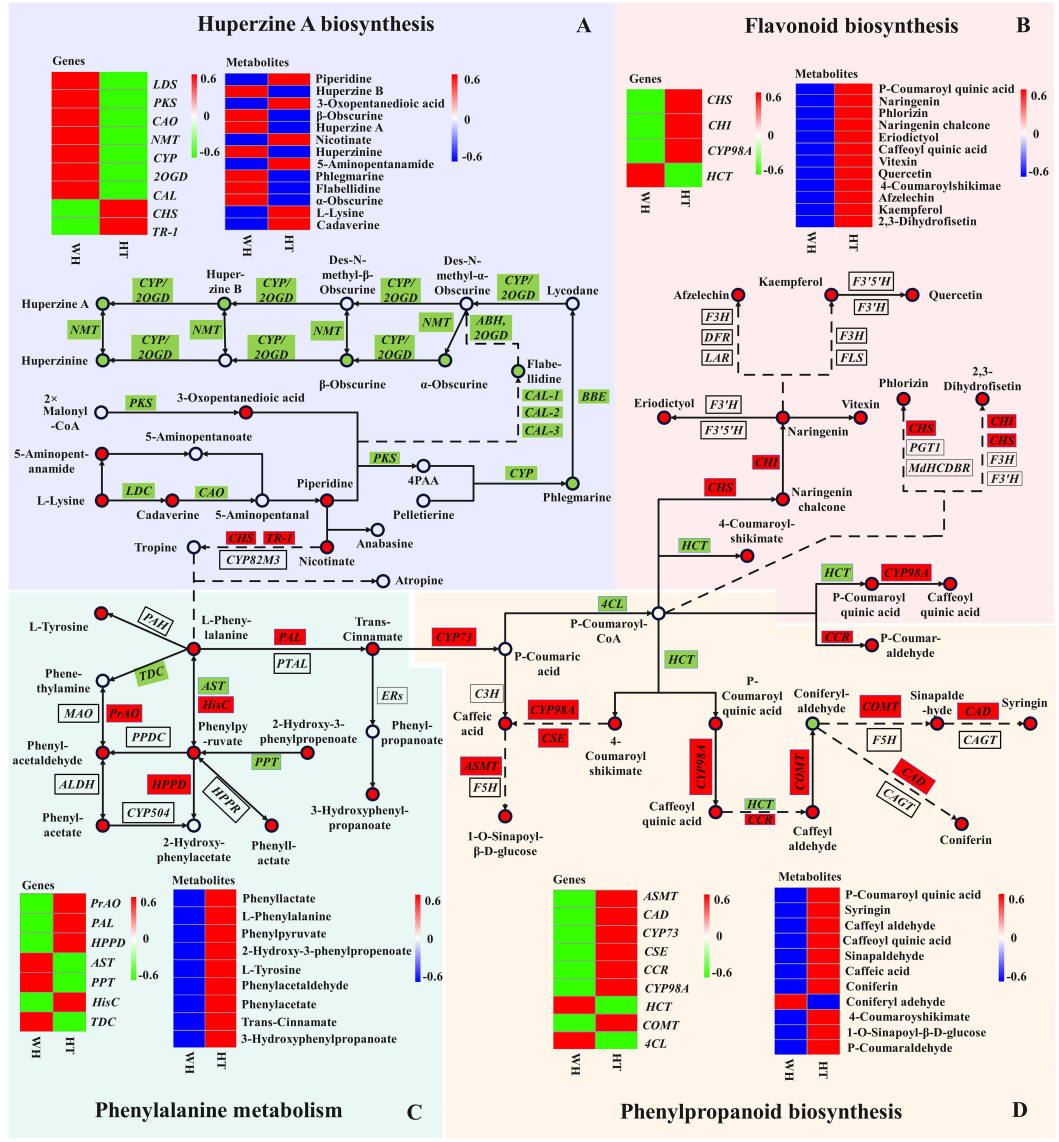
Joint pathway diagram of HupA biosynthesis and antioxidant mechanism in *H. serrata*. Pattern **(A)** represents the HupA biosynthesis, Pattern **(B)** represents the flavonoid biosynthesis, Pattern **(C)** represents the phenylalanine metabolism, and Pattern **(D)** represents the phenylpropanoid biosynthesis. The circle represents the metabolites in the pathway (red indicates up-regulation; green indicates down-regulation). The rectangles represent the regulatory genes in the pathway (red indicates up-regulation; green indicates down-regulation).

The DEGs and DAMs identified in this study were performed to KEGG co-enrichment analysis. The results showed that they were all enriched in the ko00960 pathway. Therefore, we combined ko00960 with the detected DAMs to construct a possible HupA biosynthesis pathway in *H. serrata* (**Figure 6A**; **Supplementary Figure S12**). A total of 7 gene families and 12 metabolites were involved in the HupA biosynthetic pathway. The metabolomics analysis revealed that the initial metabolites, namely l-Lysine, cadaverine, and piperidine, involved in HupA biosynthesis were up-regulated in HT. However, the downstream metabolites starting from phlegmarine were all down-regulated. This may be attributed to the downregulation expression of related downstream genes in HT, such as *PKS*, *CAL, 2OGD*, etc. Furthermore, the inhibition of the reaction between 3-Oxopentanedioic acid and piperidine to form 4PAA was also observed due to the down-regulation of *PKS* expression in HT. Consequently, the upstream product l-Lysine is accumulated in HT. Then, excessive l-Lysine further generated 5-Aminopentanamide, resulting in a lower content of HupA in HT. In WH, however, these DAMs and related genes were all upregulated, including downstream genes (*CYP, 2OGD*, and *NMT*).

Recently, *CAL-1* and *CAL-2* have been confirmed to be crucial genes in phlegmarine scaffold formation for HupA biosynthesis (Nett et al., 2023), which can catalyze piperidine and 3-Oxoglutarate to produce a very important 16-carbon scaffold and then generate flabellidine. Flabellidine would directly participate in the downstream biosynthesis reaction of HupA. In our study, 11 differentially expressed *CAL* genes were also detected, which were upregulated expression in WH (**Figure 5A**; **Supplementary Figure S12**).

### 3.8 Combined transcriptome and metabolome analysis of antioxidant mechanism in *H. serrata*

Herbs are characterized by a high content of bioactive substances, and phenolic compounds such as phenolic acids and flavonoids have antioxidant activity (Foss et al., 2022). In the metabolome analysis, 86.8% of flavonoids and 75.1% of phenolic acids were up-regulated compounds in HT. And these compounds have a significant correlation with antioxidant activity (**Supplementary Table S2**). Then, we performed KEGG enrichment analysis on all flavonoid and phenolic acid metabolites, and the results showed that the three most significantly enriched secondary metabolic pathways were flavonoid biosynthesis (ko00941), phenylpropanoid biosynthesis (ko00940) and phenylalanine metabolism (ko00360) (**Supplementary Figure S13**).

Flavonoids and phenolic acids are mostly produced by phenylpropanoid and flavonoid biosynthetic pathways. In the flavonoid and phenylpropanoid biosynthesis pathway (**Figures 6B**, **D**), a total of 20 metabolites and 11 genes were involved. The metabolites with the most significant differences were p-Coumaroyl quinic acid, caffeoyl quinic acid, 4-Coumaroylshikimate and 2,3-Dihydrofisetin, with log_2_FC of 8.48, 8.84, 8.69 and 8.00, respectively. In addition, CHI and CHS catalyze the initiation reaction of the flavonoid biosynthetic pathway, and their log_2_FC values are large at 12.16 and 14.47, respectively. Therefore, CHS and CHI may be rate-limiting enzymes. The key genes in the phenylpropanoid biosynthesis pathway are the differential genes *HCT* and *CYP98A* that catalyze key metabolites. Their log_2_FC values were -11.97 and 13.76, respectively. Overall, most of the genes and metabolites were up-regulated in HT, indicating that a large amount of flavonoids and phenolic acids were synthesized and accumulated in HT. Therefore, the high antioxidant activity of HT may be related to the accumulation of flavonoids and phenolic acids.

In the phenylalanine metabolism pathway (**Figure 6C**), 9 DAMs were detected, of which 5 were phenolic acids and all of them were up-regulated in HT. The largest differential metabolite on this pathway was 2-Hydroxy-3-phenylpropenoate, with a log_2_FC value of 8.54. It indicated that 2-Hydroxy-3-phenylpropenoate may be the most critical differential metabolite. In the transcriptome analysis, the most significant DEGs in this pathway were *PrAO*, *PAL* and *AST*, and their log_2_FC values were 17, 16.69 and 15.33, respectively. In addition, phenylalanine is an important precursor for the synthesis of phenolic compounds. Moreover, phenylalanine can react with tropine to enter the HupA biosynthetic pathway. It is suggested that the biosynthesis of HupA is likely to be influenced by antioxidant-related metabolic pathways, and the content of HupA in *H. serrata* may be related to antioxidant capacity.

## 4 Discussion

Based on the molecular mechanism of the biosynthetic pathway of active ingredients in medicinal plants, the utilization of genetic engineering technology for biosynthesis is considered one of the most effective and promising approaches to increase the content of plant secondary metabolites and address the issue of drug sources (Jiang et al., 2021). In recent years, significant progress has been made in the research and biosynthesis of compounds such as artemisinin, paclitaxel, and others (Lu et al., 2022; Zhao et al., 2022). Currently, the genetic background of *H. serrata* is still unclear, and the genomic data is very limited. It is difficult to identify and isolate key enzyme genes using traditional methods. Therefore, in this study, we conducted integrative analysis of high-throughput sequencing transcriptomics and widely targeted metabolomics for *H. serrata* thallus (HT) and wild *H. serrata* (WH). The molecular mechanism of HupA biosynthesis in *H. serrata* was analyzed. In addition, the antioxidant mechanism in *H. serrata* was analyzed by antioxidant and KEGG co-enrichment analysis. It was also evaluated that HT has good pharmacodynamic value, which has strong antioxidant activity, can scavenge free radicals in human body and is beneficial to human health.

Transcriptome sequencing can reflect the overall feature of gene expression regulation in the biological process (Wang et al., 2018). Through cluster analysis of differential transcriptome, many genes involved in the HupA synthesis pathway were identified in this study, including *CAO*, *PKS*, *LDC*, *2OGD*, *CYP*, *NMT* and *CAL*. Among them, LDC, CAO and PKS were consistent with the results of previous studies that identified them as candidate enzymes responsible for the precursor of HupA biosynthesis (Yang et al., 2017). And *2OGD*, *CYP*, *NMT* and *CAL* were also verified as genes related to the synthesis of HupA, among which *CAL* was recently identified as a very important alkaloid biosynthetic enzyme in *P. tetrastichus* (Nett et al., 2021; Li et al., 2022). And the largest DEGs (*CAO*, *CYP*, *2OGD* and *CAL*) might be the important rate-limiting enzymes and key enzymes for HupA synthesis. Meanwhile, the TFs C2H2 and PHD would play key role in the transcriptional regulation of secondary metabolite biosynthesis and response to various stresses (Wang et al., 2019, Wang et al, 2021).

Metabolites are usually considered as a bridge between genotype and phenotype, and changes in metabolite levels can directly reveal the function of genes, thus revealing biochemical and molecular mechanisms more effectively (Shen et al., 2023). In this study, a total of 1374 metabolites were identified in WH and HT. In addition, 15 metabolites were reported to be involved in the biosynthesis of HupA. Among them, 12 metabolites were detected in our metabolomics data, indicating that our metabolomics results are very reliable (Li et al., 2022). It also confirmed that HT could produce HupA. And it has the potential to become a substitute resource for WH. In addition, we found that piperidine and nicotinate reactions can participate in the phenylalanine biosynthetic pathway, and the HupA biosynthetic pathway is linked to the antioxidant metabolic pathway. In the antioxidant metabolic pathway, a total of 29 DAMs were detected, of which 28 metabolites were up-regulated in HT, indicating that the genes related to antioxidant metabolism in HT was activated and accumulated a large number of antioxidant compounds. This is consistent with the results of antioxidant experiments.

Through the analysis of metabolites and genes integration on the HupA biosynthetic pathway, three key *CAO* genes (*Cluster-33415.1*, *Cluster-37503.0*, and *Cluster-42725.0*) were found in the upstream of the pathway. Their |log_2_FC| were 10.47, 10.88 and 11.86, respectively. The key upstream metabolite is 5-aminopentanamid, and its log_2_FC was to 13, which was much larger than other metabolites in the upstream. However, l-Lysine did not further generate HupA after generating 5-aminopentanamid. It can be seen that the formation of 5-aminopentanamid in *H. serrata* is the main reason for its low HupA content. Therefore, this suggests that the accumulation of HupA in HT can be improved by regulating the expression of *CAO* gene. In addition, the |log_2_FC| of *CYP* (*Cluster-13402.0*) and *2OGD* (*Cluster-13387.0*) were 15.69 and 17.02, respectively. Their catalytic products huperzine B, hupA and phlegmarine were the metabolites with the largest fold change. Therefore, *CYP* gene (*Cluster-13402.0*) and *2OGD* gene (*Cluster-13387.0*) might be the key enzyme genes in the HupA synthesis pathway, and their products huperzine B, hupA and phlegmarine are the key metabolites in this pathway. These results further indicated that HT is an important experimental material for revealing the biosynthesis mechanism of HupA. It also laid an important foundation for cloning and identifying key enzyme genes in the HupA biosynthesis pathway.

In addition, the potential antioxidant regulatory mechanisms in *H. serrata* were investigated in this study through KEGG co-enrichment analysis. The results showed that phenylalanine, phenylpropanoid and flavonoid biosynthesis pathways were enriched as antioxidant metabolic pathway. A total of 18 DEGs and 29 DAMs were enriched in the antioxidant pathway, of which 28 DAMs were up-regulated metabolites in HT. It indicated that the content of antioxidant-related compounds accumulated in HT was higher than that in WH, which is consistent with the results of antioxidant experiments. By analyzing the log_2_FC values, the most significantly DEGs were *PrAO*, *PAL* and *AST*, respectively, of which *PAL* was the initiation and rate-limiting enzyme (Ge et al., 2023). In addition, it was analyzed that *CHI* and *CHS* are key enzymes in the flavonoid biosynthesis pathway, which is consistent with the report of Jiang et al (Jiang et al., 2020). These results confirmed that *PAL*, *CHI* and *CHS* are essential genes in the phenylalanine biosynthesis pathway. Additionally, we discovered that *PrAO* and *AST* are also essential genes in this pathway in *H. serrata*. Furthermore, it was found that p-Coumaroyl-CoA, caffeoyl quinic acid, *CHI*, and *CHS* were identified as key metabolites and key genes in the antioxidant pathway, which aligns with previous reports (Liu et al., 2022; Shu et al., 2023). Moreover, this study revealed that p-Coumaroyl quinic acid, 4-Coumaroylshikimate, 2,3-Dihydrofisetin, *HCT*, and *CYP89A* are newly discovered crucial metabolites and crucial genes. Considering that HT can produce HupA with lower cytotoxicity and higher antioxidant activity, it could be a promising and safe alternative medicinal resource for drug development and clinical application.

## 5 Conclusions

In this study, we comprehensively analyzed the global changes in the transcriptome and metabolome of wild *H. serrata* and *H. serrata* thallus. The cluster analysis of DEGs identified the enzyme genes involved in the HupA biosynthesis pathway as *LDC*, *CAO*, *PKS*, *CYP*, *2OGD*, *NMT* and *CAL*, among which *CYP* (*Cluster-13402.0*) and *2OGD* (*Cluster-13387.0*) might be the key genes. KEGG enrichment and log_2_FC analysis showed that *PrAO*, *AST*, *HCT*, *CYP98A*, *CHS* and *CHI* were key enzyme genes in the antioxidant pathway. DAMs analysis identified 12 and 29 metabolites involved in HupA and antioxidant biosynthetic pathways, respectively. Furthermore, KEGG pathway enrichment analysis confirmed that the pathway involved in HupA biosynthesis was ko00960, and the enriched antioxidant pathways were ko00360, ko00941 and ko00940. In addition, the antioxidant analysis showed that the antioxidant activity of HT was stronger than that of WH, indicating that HT can scavenge free radicals in human body and is beneficial to human health. Therefore, the results of this study not only provide new insights for further analysis of the biosynthesis mechanism of HupA. This study also confirmed that HT has the potential to replace WH producing of HupA, especially through genetic engineering modification in the future.

## Data availability statement

The original contributions presented in the study are publicly available. This data can be found here: https://www.ncbi.nlm.nih.gov/sra/PRJNA1106239.

## Author contributions

HW: Data curation, Formal analysis, Methodology, Writing – original draft. YS: Writing – review & editing. FZ: Supervision, Writing – review & editing. SY: Visualization, Writing – review & editing. YC: Supervision, Writing – review & editing. HY: Supervision, Visualization, Writing – review & editing. XL: Conceptualization, Funding acquisition, Resources, Supervision, Writing – review & editing.
