# Supplementary figures and images for: Combined transcriptome and widely targeted metabolome analysis reveals the potential mechanism of HupA biosynthesis and antioxidant activity in Huperzia serrata
Source: Front Plant Sci. 2024 Jun 17;15:1411471. doi: 10.3389/fpls.2024.1411471 (PMC11215074; doi:10.3389/fpls.2024.1411471)

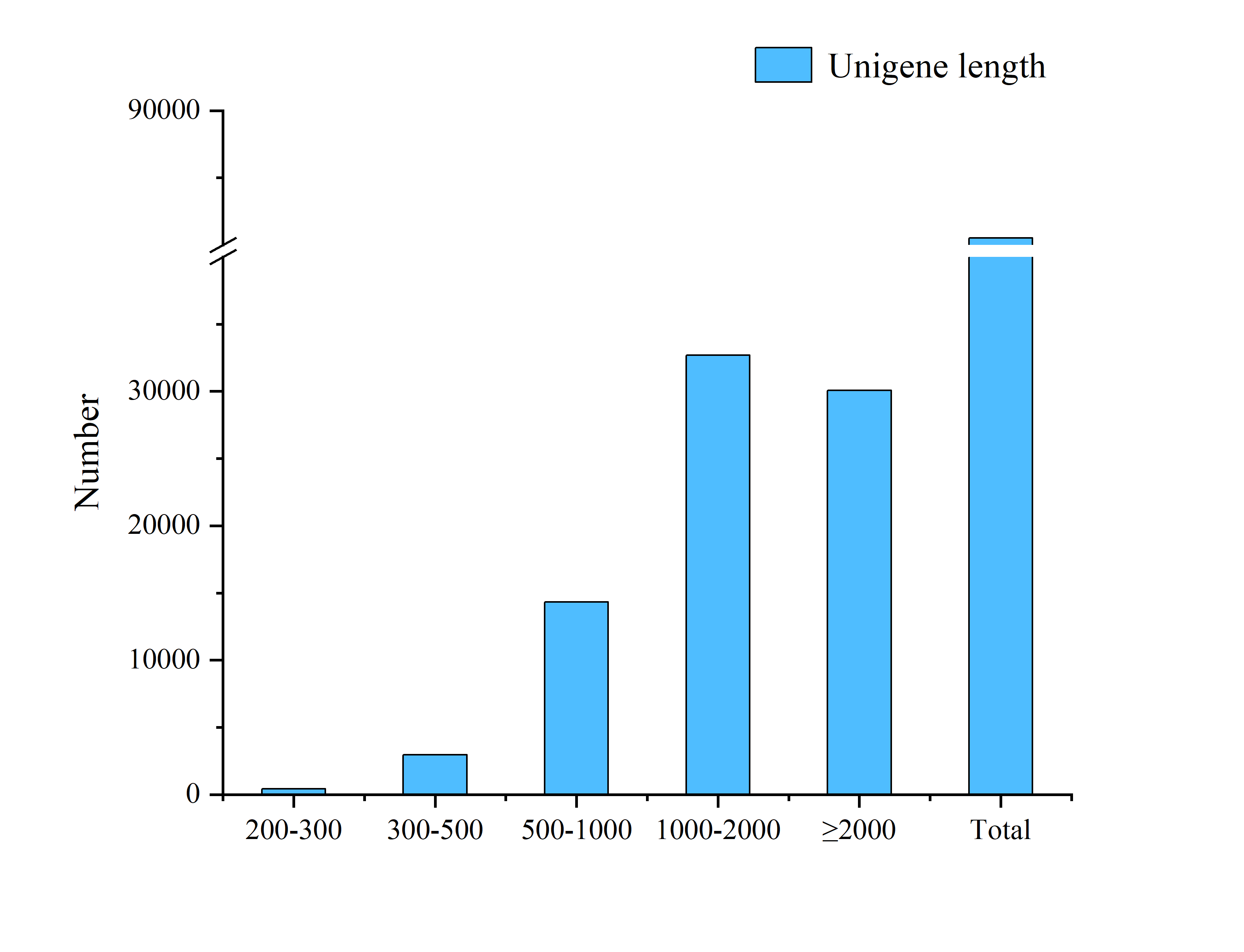

Supplement: Supplementary file 1 [file DataSheet_3.zip › supplementary figure/Fig. S1/Fig. S1.tif]

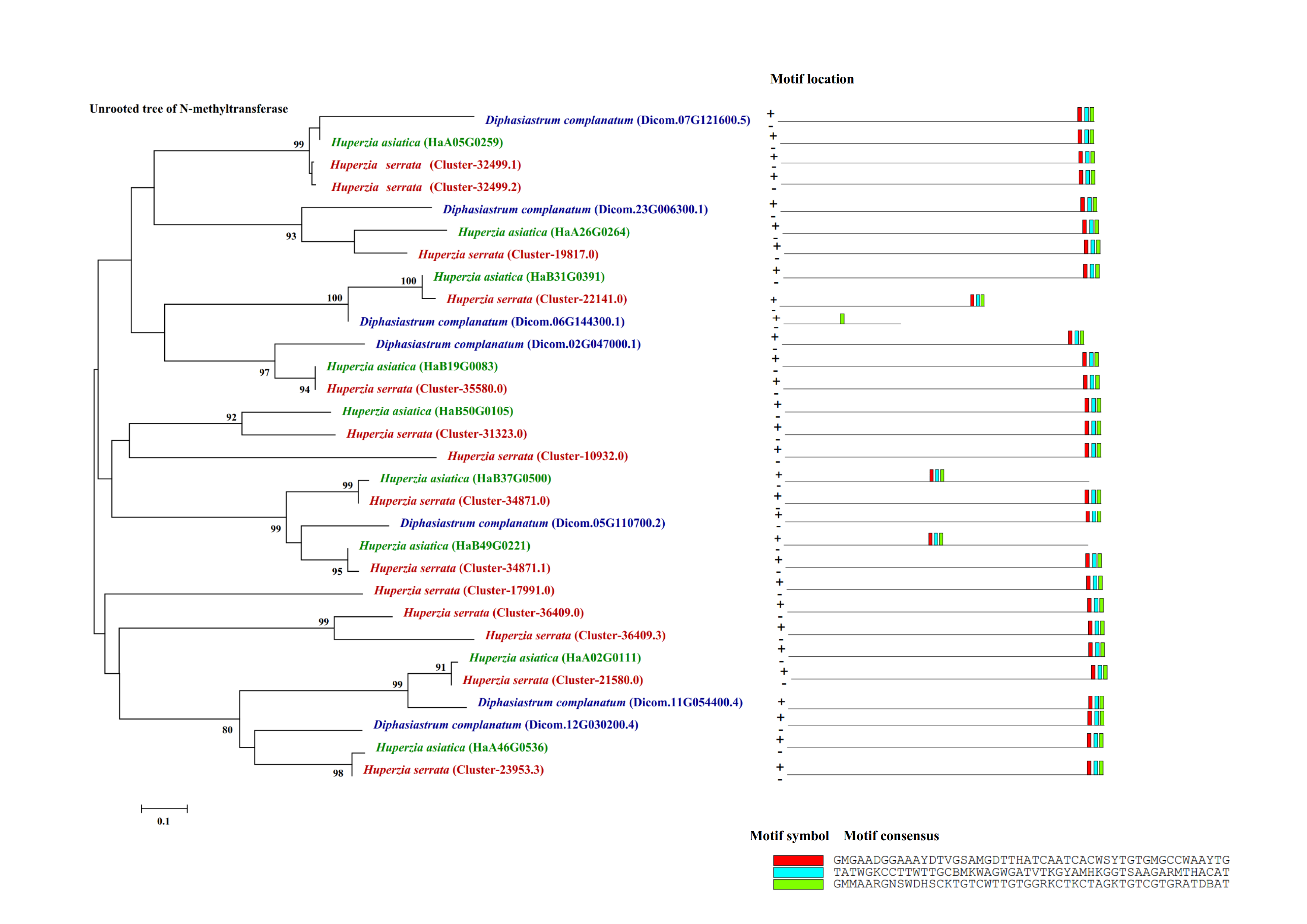

Supplement: Supplementary file 1 [file DataSheet_3.zip › supplementary figure/Fig. S10/Fig. S10.tif]

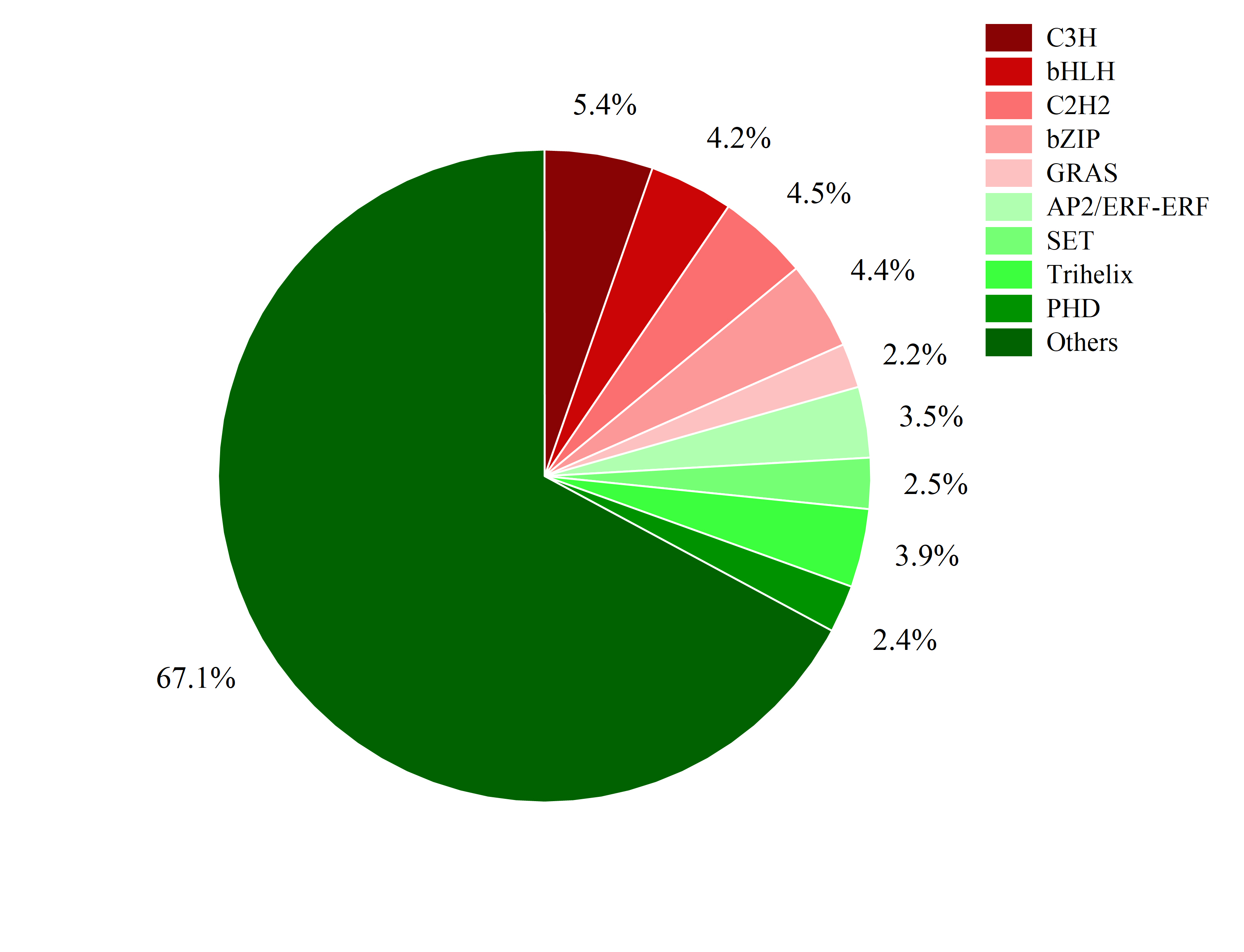

Supplement: Supplementary file 1 [file DataSheet_3.zip › supplementary figure/Fig. S11/Fig. S11.jpg]

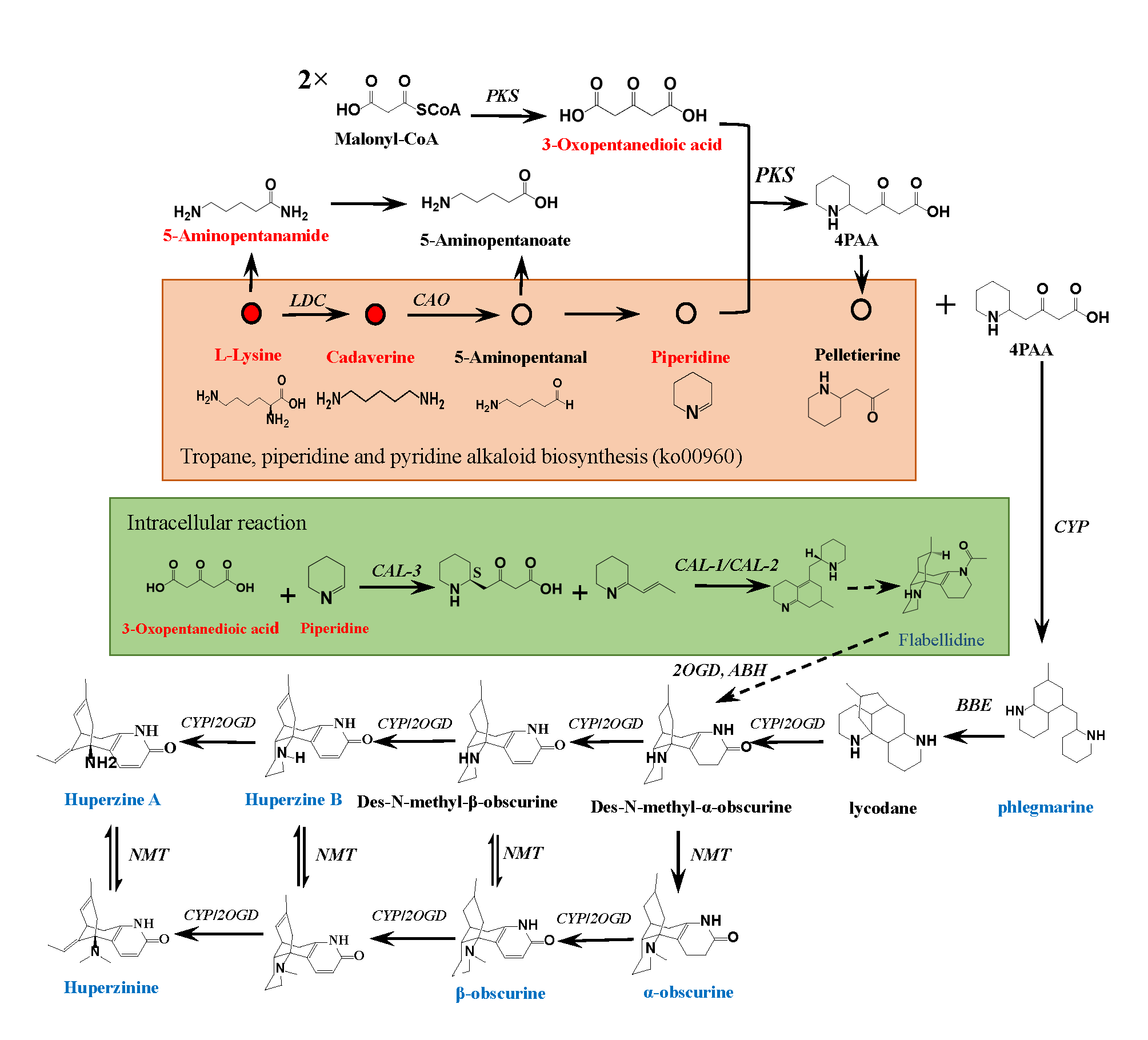

Supplement: Supplementary file 1 [file DataSheet_3.zip › supplementary figure/Fig. S12/Fig. S12.tif]

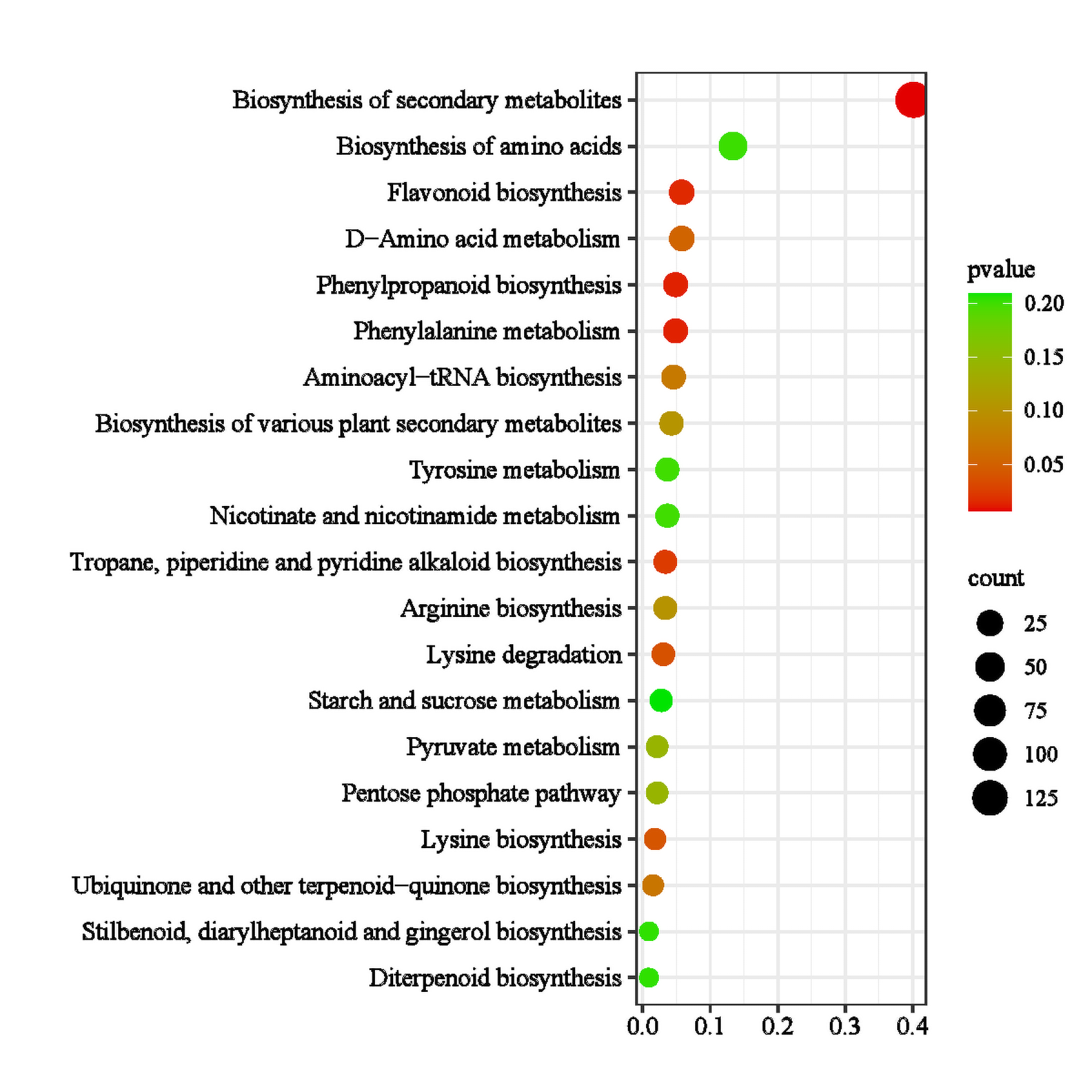

Supplement: Supplementary file 1 [file DataSheet_3.zip › supplementary figure/Fig. S13/Fig. S13.tif]

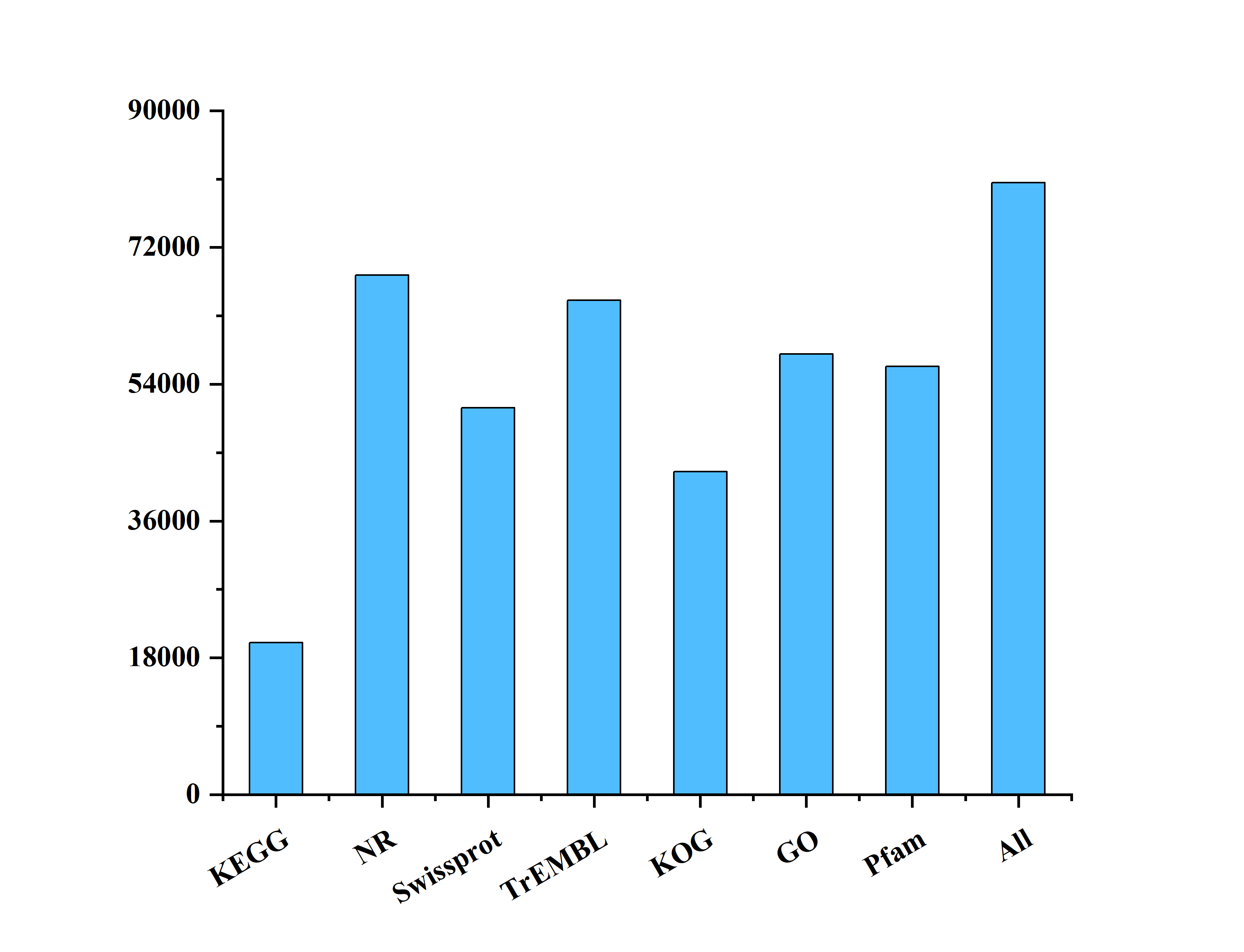

Supplement: Supplementary file 1 [file DataSheet_3.zip › supplementary figure/Fig. S2/Fig. S2.tif]

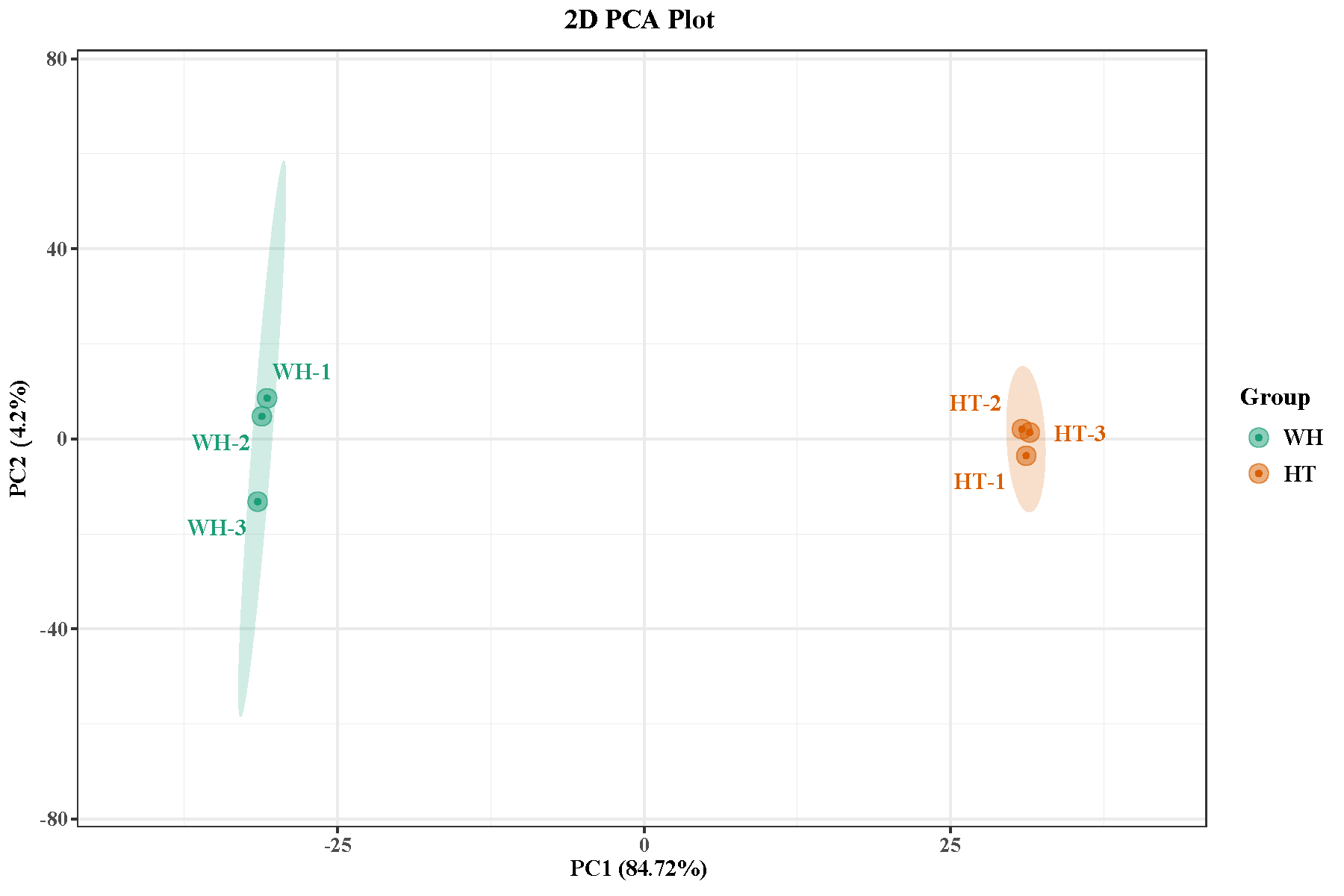

Supplement: Supplementary file 1 [file DataSheet_3.zip › supplementary figure/Fig. S3/Fig. S3.tif]

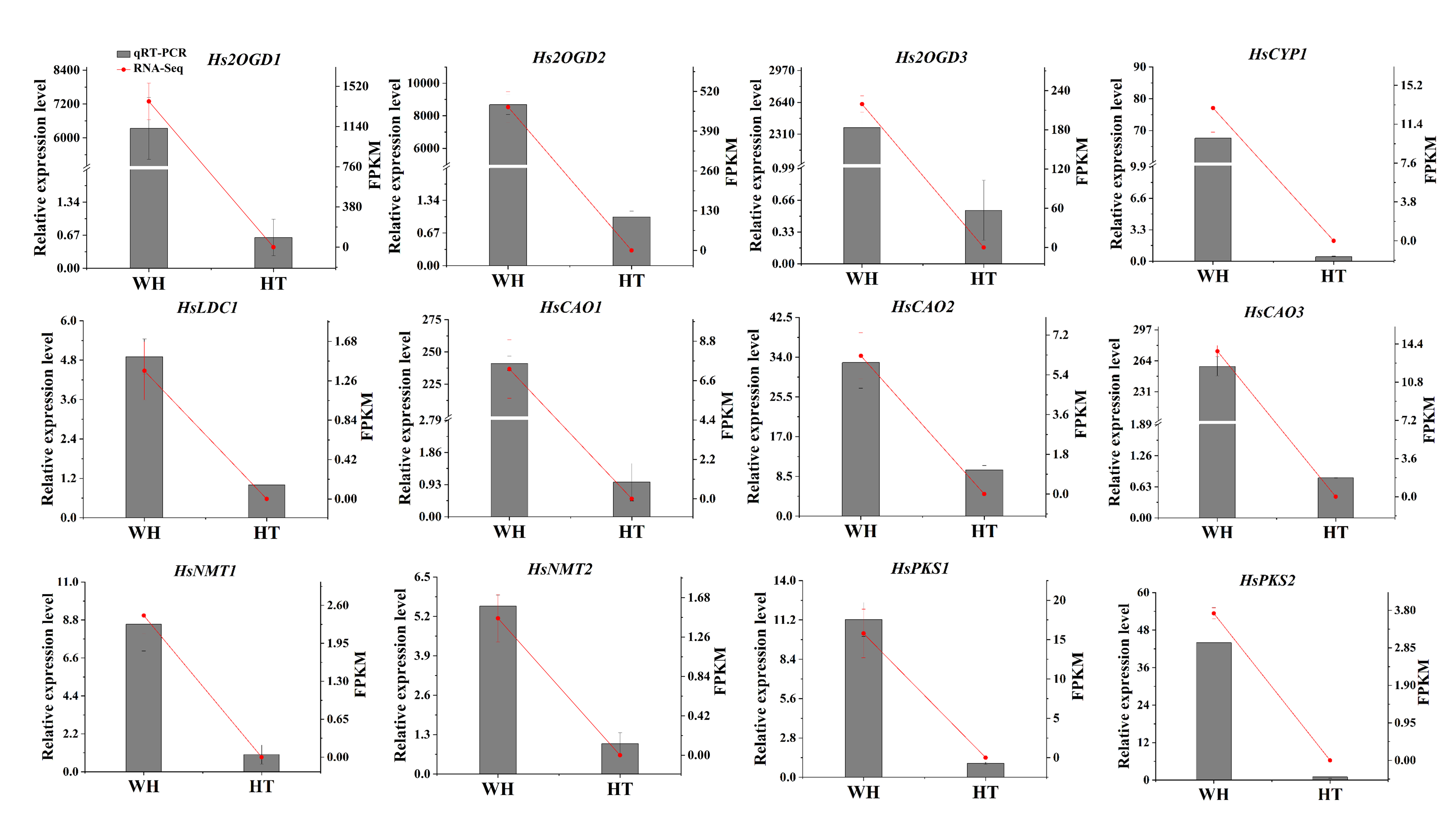

Supplement: Supplementary file 1 [file DataSheet_3.zip › supplementary figure/Fig. S4/Fig. S4.tif]

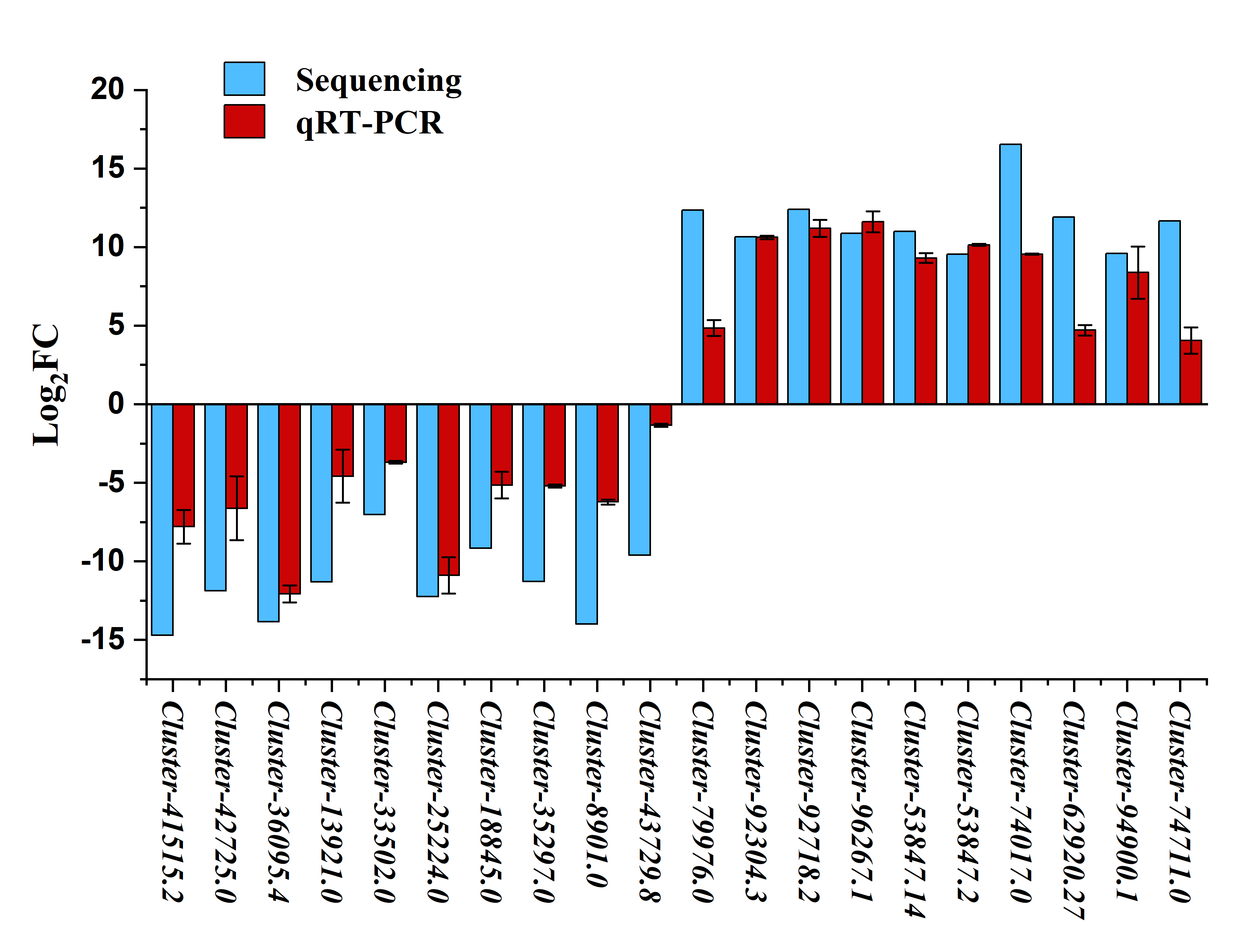

Supplement: Supplementary file 1 [file DataSheet_3.zip › supplementary figure/Fig. S5/Fig. S5.tif]

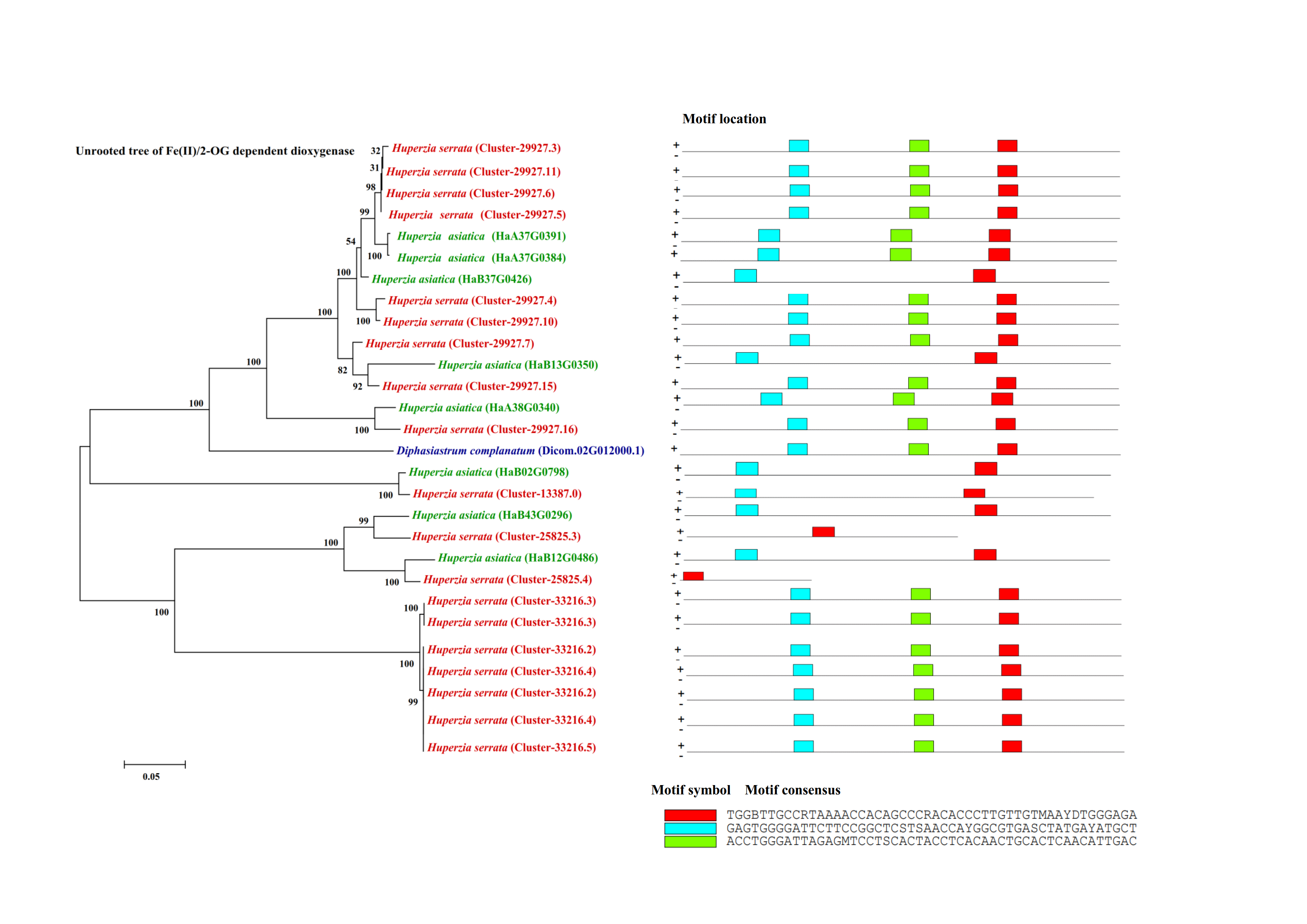

Supplement: Supplementary file 1 [file DataSheet_3.zip › supplementary figure/Fig. S6/Fig. S6.tif]

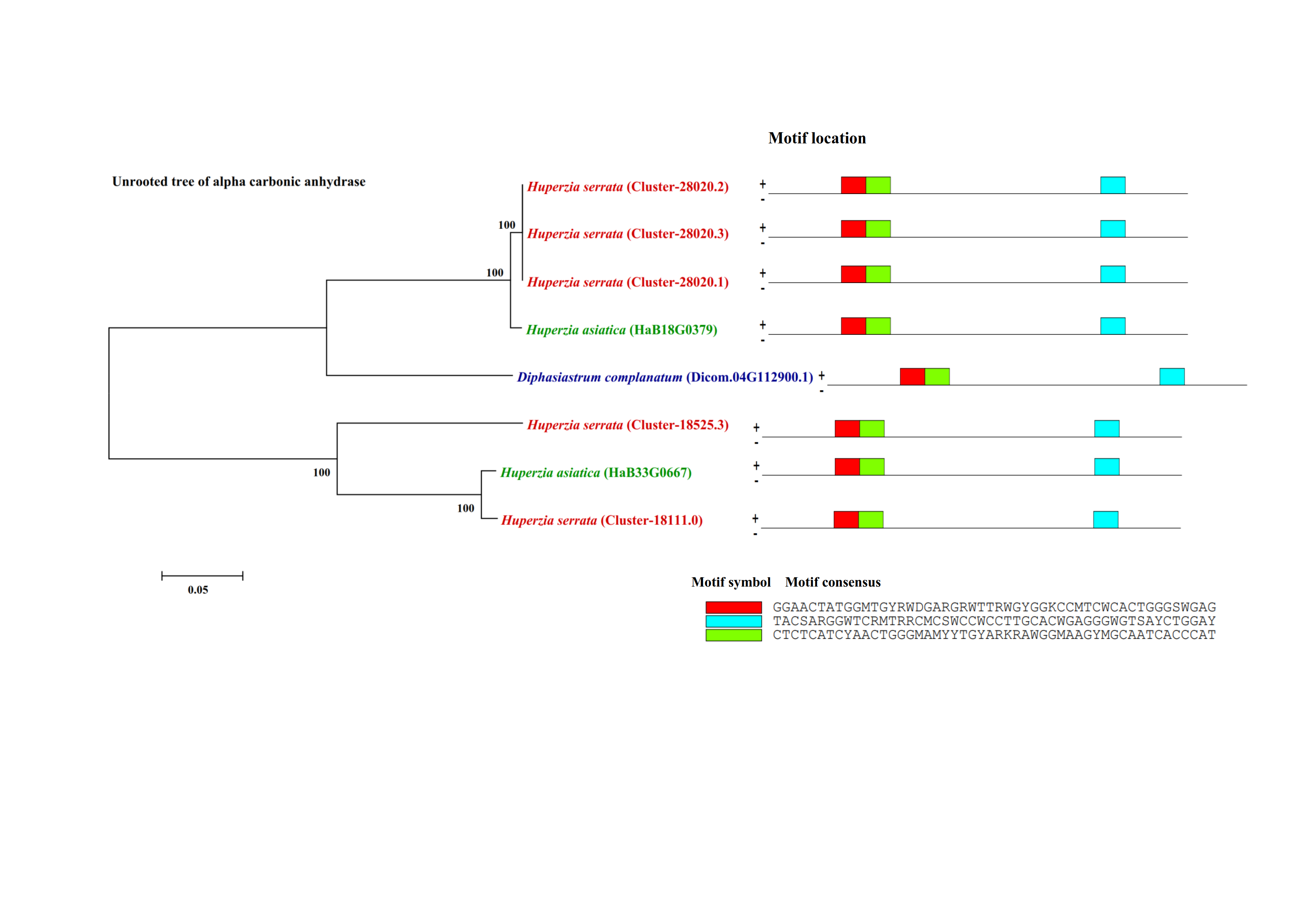

Supplement: Supplementary file 1 [file DataSheet_3.zip › supplementary figure/Fig. S7/Fig. S7.tif]

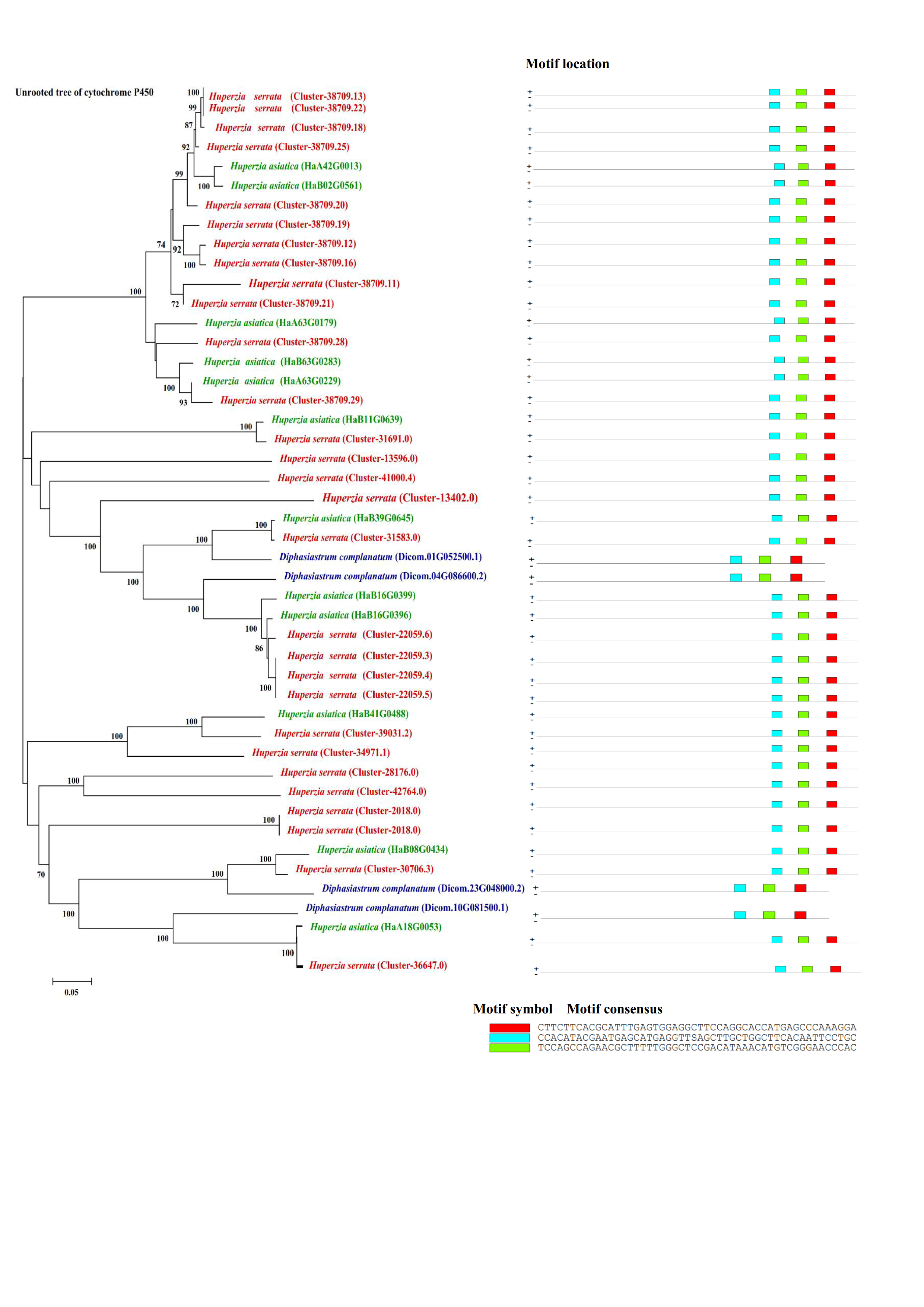

Supplement: Supplementary file 1 [file DataSheet_3.zip › supplementary figure/Fig. S8/Fig. S8.tif]

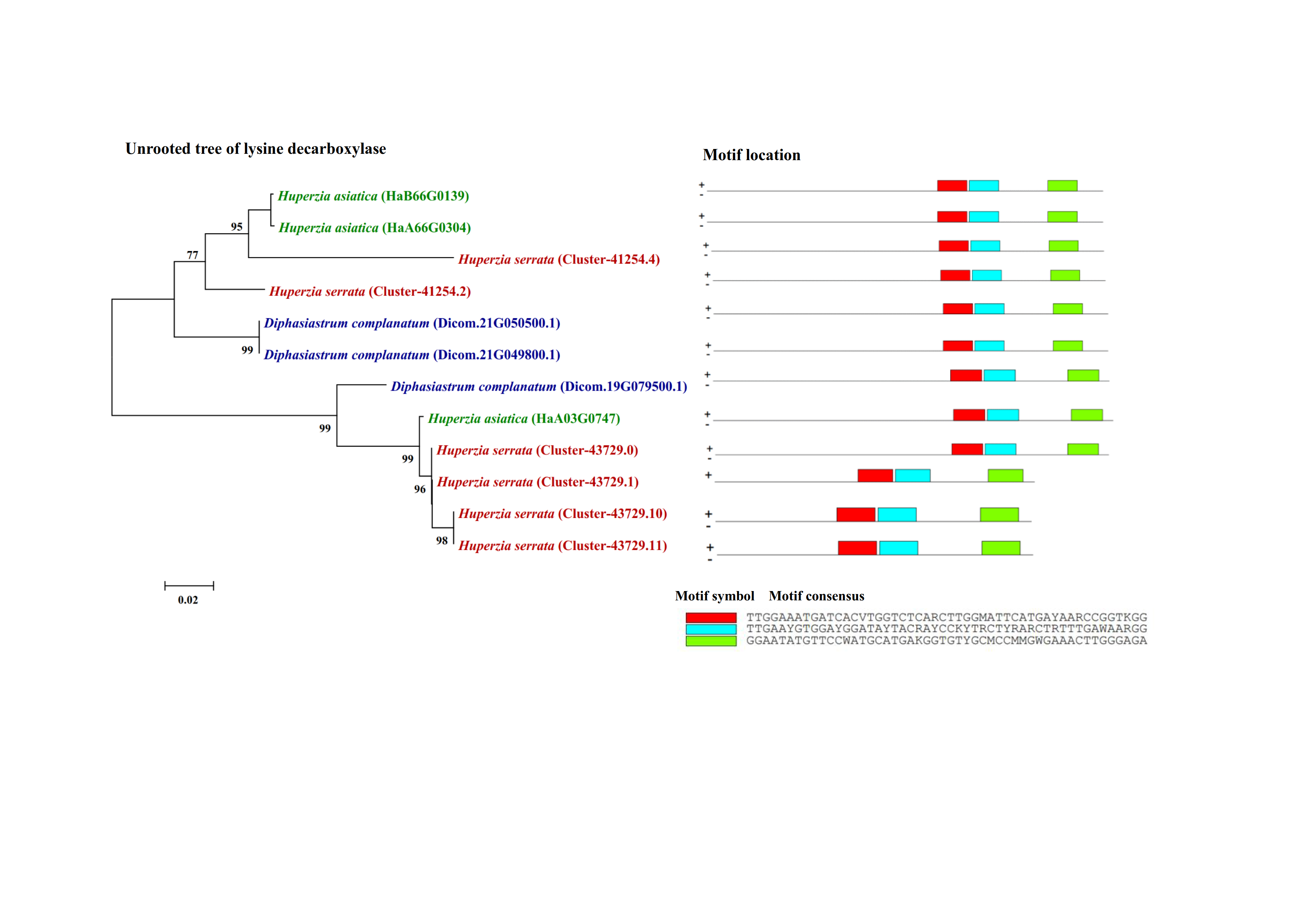

Supplement: Supplementary file 1 [file DataSheet_3.zip › supplementary figure/Fig. S9/Fig. S9.tif]
